# Mechanistic Insights of Chelator Complexes with Essential Transition Metals: Antioxidant/Pro-Oxidant Activity and Applications in Medicine

**DOI:** 10.3390/ijms23031247

**Published:** 2022-01-23

**Authors:** Viktor A. Timoshnikov, Olga Yu. Selyutina, Nikolay E. Polyakov, Victoria Didichenko, George J. Kontoghiorghes

**Affiliations:** 1 Institute of Chemical Kinetics & Combustion, 630090 Novosibirsk, Russia; olga.gluschenko@gmail.com (O.Y.S.); polyakov@kinetics.nsc.ru (N.E.P.); 2 Postgraduate Research Institute of Science, Technology, Environment and Medicine, Limassol 3021, Cyprus; vicdidichenko@outlook.com

**Keywords:** chelators, transition metals, reactive oxygen species, antioxidant activity, pro-oxidant activity, alpha-ketohydroxypyridines, hydroxypyridones, anthraquinones, thiosemicarbazones, ascorbic acid

## Abstract

The antioxidant/pro-oxidant activity of drugs and dietary molecules and their role in the maintenance of redox homeostasis, as well as the implications in health and different diseases, have not yet been fully evaluated. In particular, the redox activity and other interactions of drugs with essential redox metal ions, such as iron and copper, need further investigation. These metal ions are ubiquitous in human nutrition but also widely found in dietary supplements and appear to exert major effects on redox homeostasis in health, but also on many diseases of free radical pathology. In this context, the redox mechanistic insights of mainly three prototype groups of drugs, namely alpha-ketohydroxypyridines (alpha-hydroxypyridones), e.g., deferiprone, anthraquinones, e.g., doxorubicin and thiosemicarbazones, e.g., triapine and their metal complexes were examined; details of the mechanisms of their redox activity were reviewed, with emphasis on the biological implications and potential clinical applications, including anticancer activity. Furthermore, the redox properties of these three classes of chelators were compared to those of the iron chelating drugs and also to vitamin C, with an emphasis on their potential clinical interactions and future clinical application prospects in cancer, neurodegenerative and other diseases.

## 1. Introduction

There is an increased interest for the study of free radical reactions in biological systems and their role in health and disease, which has continued for more than 40 years [1,2,3,4,5]. Redox activity is also of interest in many areas of medicine, such as pharmacology, toxicology and metabolism and also in many biological processes, including energy transduction and the circadian rhythm [1,2,3,4,5,6,7,8]. Furthermore, in each year new, free radical mechanisms are discovered, and the schemes of metabolic pathways involving free radicals are supplemented and increase in numbers. For example, there is an increased interest following the discovery of a new mechanism of programmed cell death involving iron called ferroptosis [7,8,9,10,11,12,13]. However, despite increased research activity in this direction, all radical processes occurring within the cell during ferroptosis have not yet been fully investigated or clarified [14,15]. Nevertheless, the data obtained so far on ferroptosis allowed a new look at the pathogenesis of various diseases, including Alzheimer’s disease, atherosclerosis, cancer, etc. [15,16,17]. It should be noted that in all of the above pathologies and the overwhelming majority of other free radical processes, transition metal ions and especially iron and copper, play a very important role. Under normal conditions, the catalytic oxidative function of these transition metal ions is balanced by natural antioxidant mechanisms. However, due to various exogenous and endogenous factors, the redox balance can be disturbed, which leads to the uncontrolled generation of free radicals including reactive oxygen species (ROS) [18,19,20].

One of the earliest and most commonly used methods of treating metal-dependent pathologies was the use of chelating drugs, e.g., deferiprone (L1), deferasirox and deferoxamine, in iron overloading conditions, where a chelator can bind to metal ions and remove or displace them from the site of toxicity [3,21,22,23,24]. However, it was also found that other chelators with increased lipophilicity and other specific chelating properties can bind to transition metals, increase ROS and cause cancer cell damage, with high potential for development as anticancer drugs, e.g., omadine and triapine [25,26,27,28]. In this context and following the increased interest in the field of free radical processes, the number of publications devoted to the synthesis and development of new chelating agents and their effect on redox processes in the human body and new possible applications in medicine has grown.

In this review we will examine the effects of three main chelator groups namely the alpha-ketohydroxypyridines (KHP) or alpha-hydroxypyridones, anthraquinones, and thiosemicarbazones (TSCs), which have different redox properties. Despite the fact that these compounds and their chelating activity were discovered back in the 20th century, interest in them has not subsided to this day [29,30,31]. New chelating agents are being synthesized and the possibility of their application in various fields of medicine, including redox activity and other properties are being actively investigated. It should be noted that this review focuses on the physicochemical properties of chelators, their redox activity, the effect on the redox properties of their metal complexes, as well as their potential therapeutic use. In addition, the role of vitamin C, a natural antioxidant/chelator in the generation and inhibition of ROS with the participation of chelate complexes of transition metals will also be discussed.

## 2. The Role of Essential Transition Metals in Living Systems

The most common transition metals in living systems are iron and copper. The regulation of the absorption, transport, storage and utilization of iron in humans is primarily controlled by a number of specific regulatory proteins and transcription factors, for example hemoglobin, transferrin, ferritin, ribonucleotide reductase, etc. As with other ions of essential metals, under normal conditions, iron has a specific metabolic pathway, biomolecular sites of interaction, specific tissue deposition and storage, and specific transport routes in blood and cells, as well as excretion. Most of the iron that has entered the cytoplasm of the cell (labile iron pool) is used for the synthesis of hemoglobin in erythroid cells, whereas, in non-erythroid cells, for the synthesis of DNA, RNA, and iron-containing enzymes. The remaining part of iron is stored intracellularly in a safe, polynuclear and non-toxic form in the protein ferritin [32,33,34,35].

Iron metabolic imbalances are commonly associated with a number of serious conditions, such as iron deficiency anemia, which affects between a third and a quarter of the world’s population [36]. In contrast, many hundred thousands of patients with chronic hematological and malignant diseases, including thalassemia, sickle cell anemia, hematopoietic stem cell transplantation, aplastic anemia and cancer, suffer from iron overload due to multiple blood transfusions for the treatment of refractory anemia [37,38,39]. Likewise, iron overload caused by increased absorption of dietary iron in the gastrointestinal tract in the inherited disorder of idiopathic hemochromatosis affects millions of people [40,41].

Copper also plays an important role in the biology of living organisms. This element is widely used by cells in which it can perform regulatory and catalytic functions [42,43]. Many enzymes are associated with copper, for example, ascorbate oxidase, cytochrome oxidase, tyrosinase, superoxide dismutase, dopamine hydroxylase, etc. [44,45,46,47].

Under normal physiological conditions, the level of copper in the body is well regulated by metabolic mechanisms. However, there are many diseases associated with copper imbalance and toxicity, such as Mencke’s and Wilson’s diseases. In the case of Wilson’s disease, dietary copper absorption increases which leads to its accumulation, mainly in the liver, leading to cirrhosis. Copper accumulation is also observed in the brain, kidneys and cornea, causing damage and dysfunction of these organs [48].

The main reason for the negative effect of iron and copper is their participation in oxidation-reduction (redox) reactions, as a result of which ROS are generated and produced without control. Reactive oxygen species are capable of oxidizing and damaging almost all biomolecules including lipids, sugars, amino and nucleic acids [2,4,49]. Usually, the body can cope with oxidative stress using natural antioxidants, for example alpha-tocopherol (vitamin E), ascorbic acid (vitamin C), glutathione, superoxide dismutases (SODs), etc., which are able to trapping various ROS [50,51,52]. However, in the case of a number of diseases, the body is unable to inhibit the uncontrolled generation of ROS.

It is believed that the main mechanism of iron and copper toxicity is their ability to participate in Fenton-like reactions leading to the formation of ROS, including highly toxic hydroxyl radicals (^•^OH) [53,54,55]. The mechanism of radical generation by iron and copper has been studied in detail by various physical methods, including EPR with spin traps, and is described in detail in the literature and the reaction Equations (1)–(5) [56,57,58,59].

(1) $${\mathrm{Cu}}^{2+}+H_{2}O_{2}\overset{k_{1}=460\;M^{-1}s^{-1}}{\to }{\mathrm{Cu}}^{+}+2H^{+}+{\dot{O}}_{2}^{-}$$

(2) $${\mathrm{Cu}}^{+}+H_{2}O_{2}\overset{k_{2}=<100\;M^{-1}s^{-1}}{\to }{\mathrm{Cu}}^{2+}+\dot{\mathrm{OH}}+{\mathrm{OH}}^{-}$$

(3) $${\mathrm{Fe}}^{2+}+H_{2}O_{2}\overset{k_{3}=63\;M^{-1}s^{-1}}{\to }{\mathrm{Fe}}^{3+}+\dot{\mathrm{OH}}+{\mathrm{OH}}^{-}$$

(4) $${\mathrm{Fe}}^{3+}+H_{2}O_{2}\overset{K=3.1\times 10^{-3}}{↔}{\left[{\mathrm{Fe}}^{3+}\left({\mathrm{HO}}_{2}\right)\right]}^{2+}+H^{+}$$

(5) $${\left[{\mathrm{Fe}}^{3+}\left({\mathrm{HO}}_{2}\right)\right]}^{2+}\overset{k_{4}=2.7\times 10^{-3}\;s^{-1}}{\to }{\mathrm{Fe}}^{2+}+{H\dot{O}}_{2}$$

The rate of reaction Equation (2) can vary significantly (~100–10,000 M^−1^s^−1^) depending on the temperature, ionic strength, pH, and oxygen content of the solution. This reaction is described in more detail in [59].

However, there is no consensus on the mechanism of the Fenton reaction with the participation of copper ions [56,60,61]. Some of the authors suggest that there are intermediate reactions with the formation of the Cu (III) ion as an intermediate product of reaction Equations (6)–(8), as well as the formation of hydroxyl radicals as the final product of these reactions [56,57]. There are also different opinions about the reaction rate constant regarding Equation (1).

(6) $${\mathrm{Cu}}^{+}+H_{2}O_{2}\overset{k_{5}=61{\;M}^{-1}s^{-1}}{\to }{\mathrm{Cu}}^{3+}+2{\mathrm{OH}}^{-}$$

(7) $${\mathrm{Cu}}^{3+}+{\mathrm{Cu}}^{+}\overset{k_{6}=3.5\times 10^{9}\;M^{-1}s^{-1}}{\to }2{\mathrm{Cu}}^{2+}$$

(8) $${\mathrm{Cu}}^{3+}+H_{2}O_{2}\overset{k_{7}\leq 1.3\times 10^{5}\;M^{-1}s^{-1}}{\to }{\mathrm{Cu}}^{2+}+2H^{+}+{\dot{O}}_{2}^{-}$$

Reactive oxygen species formed during the Fenton reaction can initiate more complex biochemical chain reactions, for example, the peroxidation of lipids that are components of cell membranes. In particular, the recently discovered mechanism of programmed cell death ferroptosis is based on lipid peroxidation of the cell membranes with the participation of iron ions, which leads to disruption of homeostasis, destruction of the cell membrane and ultimately cell death [62,63]. However, despite the enormous interest from the scientific community, the detailed mechanism of this reaction has not been fully understood due to the complexity of the system and the short lifetime of the intermediates formed in the course of the redox reactions [64].

## 3. The Potential Antioxidant/Pro-Oxidant Effects of Ascorbic Acid

**Figure 1:**
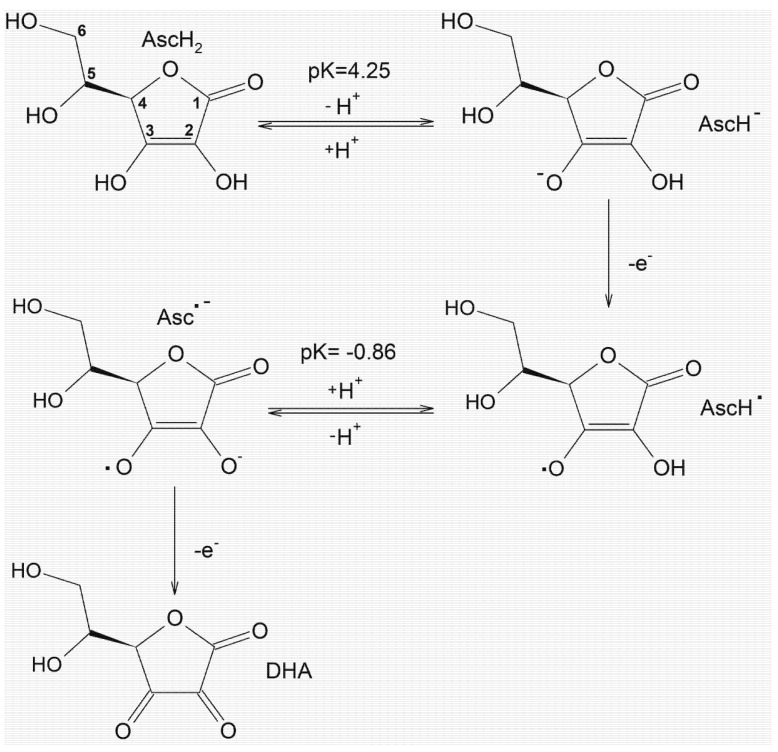
The structure of ascorbic acid (AscH_2_) and its scheme of oxidation with the main products of redox reactions namely ascorbate anion (AscH^−^), ascorbyl radical (AscH^•^), ascorbyl anion radical (Asc^•−^) and dehydroascorbic acid (DHA).

Ascorbic acid (AscH_2_) is one of the main vitamins which is not synthesized in human and other primate organisms. This compound is extremely important for the normal functioning of the body and is found in many vegetables and fruits. Ascorbic acid is mainly found in acidic conditions, while at neutral pH it is in the form of the ascorbate anion (AscH^−^). Under certain conditions AscH_2_ is capable of oxidizing, transforming first into the ascorbyl radical (AscH^•^), and then into the dehydroascorbate (DHA) (Figure 1). In addition to its nutritional value, ascorbic acid has wide medical applications, including the treatment of vitamin C deficiency, pneumonia, acute respiratory failure, multiple myeloma, metastatic colorectal carcinoma, metastatic melanoma, coronary heart disease, type 2 diabetes, dementia, Alzheimer’s disease and COVID 19 [65,66,67,68,69,70,71,72,73,74]. Ascorbic acid has a number of physicochemical and biological functions, including antioxidant, chelating, and coenzyme activities [75,76,77].

Moreover, ascorbate is also involved in tissue repair, the production of neurotransmitters and is also important for the immune system. The main function of ascorbic acid is its antioxidant activity through its ability to interact with composite radicals, restoring them as shown in the Equation (9):

(9) $${\mathrm{AscH}}_{2}\;\left({\mathrm{or}\;\mathrm{AscH}}^{-}\right)+\dot{R}\to \dot{\mathrm{Asc}}\left(\mathrm{or}\;{\dot{\mathrm{Asc}}}^{-}\right)+\mathrm{RH}$$

where $\dot{R}$ is the free radical (see Table 1).

As a powerful water-soluble antioxidant, ascorbic acid provides effective protection to cell membranes, proteins and other biomolecules from oxidation by many oxidants, including superoxide radical (O_2_^•−^), hydrogen peroxide (H_2_O_2_), ^•^OH, peroxyl radical (^•^OOH) and singlet oxygen (^1^O_2_) [2,4,78,79,80]. As a result of the redox reaction, a stable radical AscH^•^ is formed. However, under physiological conditions, this radical is deprotonated (pKa −0.86), transforming into the Asc^•−^ radical. The rate constants of the reactions with the most common radicals were previously calculated as shown in Table 1 [81].

In addition to antioxidant activity, ascorbic acid can also exhibit pro-oxidant activity [82,83,84,85,86,87,88,89]. It can react with oxidized transition metal ions such as Fe^3+^ and Cu^2+^, causing their reduction Equations (10) and (11):

(10) $${\mathrm{Fe}}^{3+}+{\mathrm{AscH}}_{2}\;\left({\mathrm{or}\;\mathrm{AscH}}^{-}\right)\to {\mathrm{Fe}}^{2+}+\dot{\mathrm{AscH}}\left(\mathrm{or}\;\dot{{\mathrm{Asc}}^{-}}\right)+H^{+}$$

(11) $${\mathrm{Cu}}^{2+}+{\mathrm{AscH}}_{2}\;\left({\mathrm{or}\;\mathrm{AscH}}^{-}\right)\to {\mathrm{Cu}}^{+}+\dot{\mathrm{AscH}}\left(\mathrm{or}\;\dot{{\mathrm{Asc}}^{-}}\right)+H^{+}$$

Further, the reduced metal ions are able to participate in the Fenton reaction Equations (2) and (3). Thus, the reactions of ascorbic acid with Fe^3+^ and Cu^2+^ ions are an additional channel for the formation of ROS. As a result, there is a significant increase in the rate of oxidation of various compounds, including many biomolecules [89,90,91,92]. In general, there is a debate in the medical community about the use of ultrahigh doses of ascorbic acid for the treatment of cancer in combination with other anticancer agents [93,94,95,96,97,98].

It should be mentioned that ascorbic acid is capable of chelating various metals. There are many studies on the influence of its metal coordination properties on important biological redox processes [99,100,101,102,103]. The equilibrium constants of ascorbic acid metal complexes are low in comparison with various chelating drugs used to treat diseases associated with metal overload [104,105]. The main chelate center of ascorbic acid is considered to be O (2) and O (3) nuclei, which are capable of forming non-covalent donor–acceptor bonds with a metal ion after deprotonation of the OH groups [99]. There are many studies of chelating complexes of ascorbic acid with iron and copper ions. Complexes with Fe^3+^ and Cu^2+^ ions are unstable and decompose due to intramolecular electron transfer within a few milliseconds with the formation of oxidation products such as DHA and Cu^+^ and Fe^2+^ ions [106,107,108]. In turn, the complexes of ascorbic acid with the Fe^2+^ ion are fairly stable, with stoichiometry 2:1 [Asc_2_Fe^2+^] [99]. As for Cu^+^, these complexes are unstable in aqueous solutions due to the high reactivity with ions, which can be oxidized to Cu^2+^ [107,109].

Despite that ascorbic acid has a relatively low affinity for metals, it can interact with other chelate complexes, affecting their redox activity and can cause either an increase or a decrease in the production of ROS. The main mechanisms of action are the reactions of electron transfer between the chelator and ascorbic acid, as well as the incorporation of ascorbate into the internal coordination sphere of the complex [74,110,111,112,113]. More details of the effects of ascorbic acid on the redox activity of chelators are discussed below.

## 4. Redox Properties and Biological Implications of Chelating Drugs

The major use of chelating drugs is the treatment of diseases associated with metal overload. These drugs are able to bind to metal ions and remove them from the body. The main requirements for clinical use of these chelators are mainly high affinity for a particular metal, bioavailability, high stability constants of the complexes and low toxicity. An important aspect of toxicity is that the resulting chelator metal complexes should not participate in redox reactions with the formation of increased ROS.

There are two main actions of chelators that can reduce oxidative stress caused by metal ions, namely the decrease in the redox potential (energetic) and binding to vacant coordination sites of a metal ion (dimensional) [114]. Another aspect of toxicity is the photostability of chelators and their metal complexes upon the formation of chelate complexes, with photosensitivity of the drug being an important parameter considered in clinical use. In this context, under the action of radiation, the chelator and/or metal is able to participate in redox reactions with the formation of ROS or toxic products [2,4,49].

**Figure 2:**
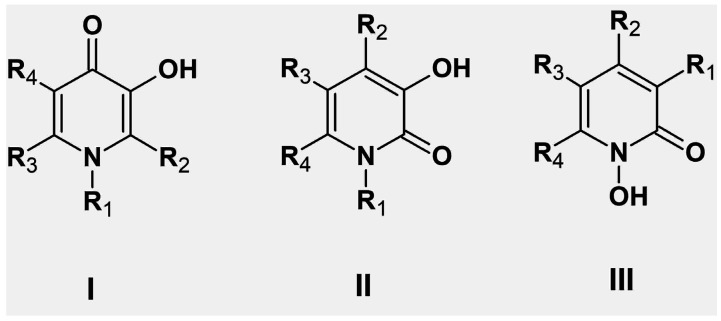
Chemical structures of alpha-ketohydroxypyridine chelators.

One of the most effective groups of iron chelators is the alpha-ketohydroxypyridines (KHP) or alpha-hydroxypyridones [115,116,117,118,119,120,121,122]. About 100 KHP have been synthesized including bidentate, tetradentate and hexadentate chelators, which have been screened in vitro, in vivo and also some in clinical trials for clinical use in the treatment of iron overload and other diseases [22,23,118,122,123,124,125,126,127]. There are three prototype structures the 3-hydroxypyrid-4-ones (I). 3-hydroxypyrid-2-ones (II) and 1-hydroxypyrid-2-ones (III). In each case the iron binding site comprises of an oxygen atom (O) and adjacent hydroxyl (–OH) group (Figure 2).

In addition to treating diseases associated with metal overload in the body, chelators are also used to treat cancer. There are many approaches to potential cancer treatment using chelators including photodynamic therapy and chemotherapy [128,129]. The chelators with anticancer activity can be divided into many categories including two main group categories according to the type of effect on the cancer cell. In one category, the effect is related to molecular insertion into DNA, thereby inhibiting the replication and repair of genetic material and the second is participation in redox reactions with the formation of ROS, which in turn oxidize and destroy DNA, proteins, and lipid membranes [130,131,132,133,134].

**Figure 3:**
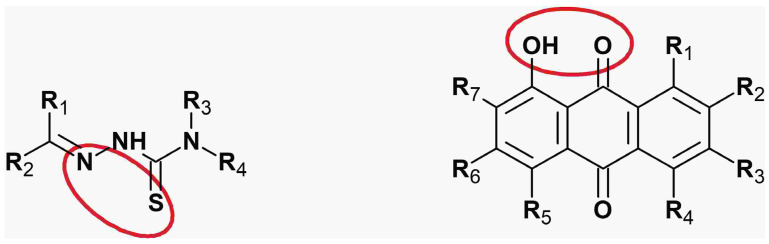
Chemical structures of thiosemicarbazones and anthraquinone-like anticancer drugs with chelating potential. The red circle marks the chelating centers of the molecules. R_n_ denotes various substituents.

There are many other groups of chelators with potential anticancer activity with different chemical structures not related to KHP. Among the most promising chelating groups of anticancer agents are the anthraquinones and TSCs (Figure 3). Nevertheless, the anticancer drug members with metal chelating potential of these two groups have a number of toxic side effects, similar to any other drug, including low selectivity for cancer cells, cardiotoxicity, hair follicle toxicity, etc. [135,136].

**Scheme 1:**
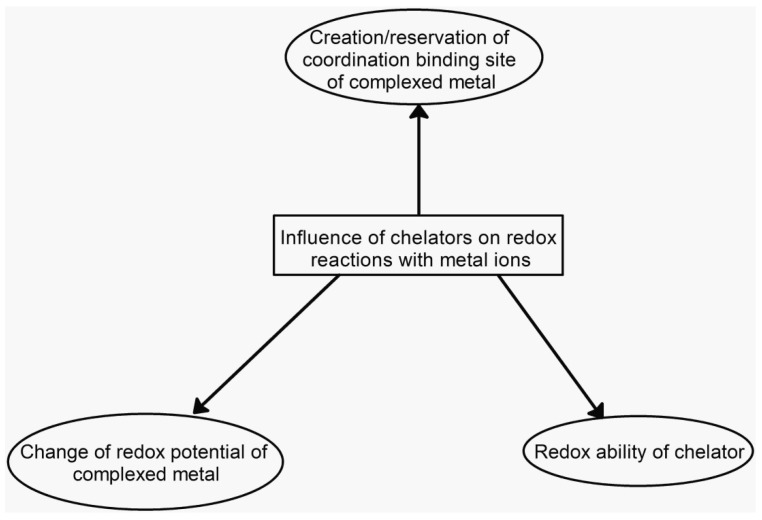
Influence of chelators on redox reactions with metal ions.

In general, chelatting drugs have different physicochemical properties that affect the course of redox reactions associated with transition metal ions. In this context, at least three separate redox related mechanisms can be distinguished, namely the ability of the chelator to participate in redox reactions, a change in the redox (electrode) potential of a complexed metal, and the creation/reservation of coordination binding site of complexed metal (Scheme 1).

It should be noted that some chelators can participate in redox reactions with the formation of ROS in the absence of metal ions. For example, most of the biomolecules in biological systems such as DNA, protein and lipids are electron donors and chelators with electron acceptor properties, which are able to enter into dark and photoinduced chemical reactions, forming ROS [2,4,49]. Among such chelators, anthraquinones can be distinguished, since they have not only electron acceptor properties, but are also capable of participating in redox reactions with complexed transition metal ions, such as iron and copper, transforming them into a more reactive state, thereby inducing the Fenton reaction Equations (1)–(8) [137]. However, some other chelators can act as radical scavengers due to certain chemical groups, e.g., –OH in the structure of the molecule. An example are phenol-containing compounds where the hydroxyl group is capable of trapping various ROS [138,139].

Another mechanism of chelators that affects the redox activity of complexed metals is the change of the redox (electrode) potential of a metal ion. It is known that metal-chelate is an effective catalyst for redox reactions if its potential lies in the range between E^0^(O_2_/O_2_^•−^), −0.33 V and E^0′^(H_2_O_2_,H^+^/HO^•^,H_2_O), +0.39 V [86,140]. Thus, if the electrode potential of the complexed metal is below −0.33 V, then the redox reaction will be energetically unfavorable. However, if the electrode potential is above +0.39 V, then the rate of redox reactions becomes diffusion controlled. In this regard, after several cycles of reactions, the redox activity of the chelate complexes decreases and the concentration of potential electron donors/acceptors in the local environment of metal-chelate also decreases [86,140]. Therefore, metal-chelate with an electrode potential ranging from −0.33 V to +0.39 V will be the most effective redox active agents.

A third mechanism influencing the redox activity of complexed metals is the creation/reservation of a coordination binding site of the complexed metal. In this case, this implies the possibility of adding hydrogen peroxide (H_2_O_2_) or another ligand to the internal coordination sphere of the complex with further formation of various ROS [86,114]. This mechanism depends on many factors, including the coordination number of the complexed metal, the density of the chelator, the number of ligands in the internal coordination sphere, the stability constants of the complex, the charge of the internal coordination sphere, pH etc. These factors cannot be considered separately, because they are interrelated with each other and affect the reactivity of the complexed metal ion.

Overall, it can be suggested that the effect of a chelator on the redox activity of a complexed metal ion is a multifactorial system consisting of several mechanisms. These mechanisms could be analyzed in detail using several groups of chelators, including the KHP, anthraquinones, and TSCs, and the possible applications and other implications in medicine.

## 5. Alpha-Ketohydroxypyridine Chelators

**Figure 4:**
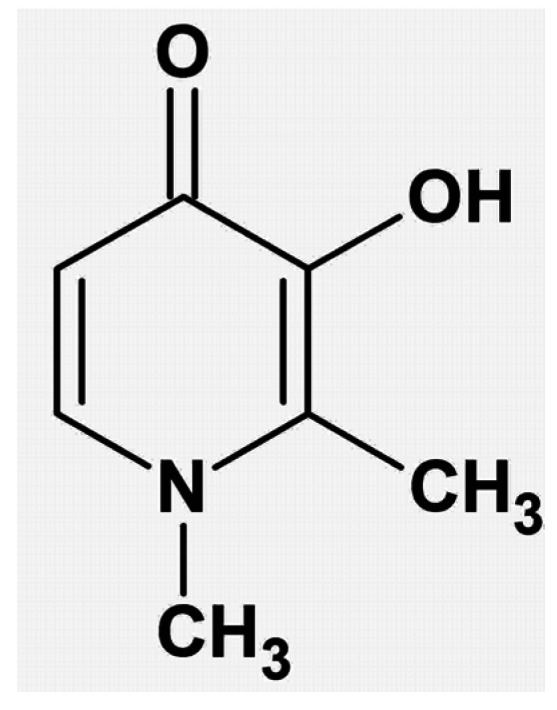
Chemical structure of deferiprone (L1). The alpha-ketohydroxy metal binding site of L1 comprises of an oxygen atom (O) and adjacent hydroxyl (–OH) group.

The alpha-ketohydroxypyridine (KHP) or alpha-hydroxypyridinone class of chelators has been designed and actively studied since the 1980s, leading to the discovery of L1 (Figure 4) [21,22,23,118,119,120,121,122,123,124,125,126,127,141]. This impetus served to develop a therapeutic direction associated with the treatment of diseases caused by an excess of metals in the body. Most of the KHP synthesized are bidentate chelators with an oxygen atom (O) and adjacent hydroxyl (–OH) group at the metal binding site with the two oxygen molecules as the electron donors for bond formation with metal ions. The great interest in this group of chelators stems from the high affinity/binding constant of iron and other metal ions, as well as low toxicity [116,118,119,120,121,122,142].

There are many potential applications in the use of KHP and their metal complexes in biology and medicine, including the treatment of diseases associated with excess of iron and other metal deposition, e.g., neurodegenerative diseases or iron metabolic imbalance [143,144,145]. The effect of KHP chelators on the redox activity of metals is of pharmacological and toxicological importance in all these diseases. In the case of iron and copper overloading diseases, KHP are required to possess antioxidant activity in order to reduce the oxidative stress caused by the uncontrolled generation of ROS in reactions involving transition metal ions as shown in the reaction Equations (1)–(8).

Despite more than 30 years of development of pharmacological research in chelation therapy for the treatment of iron overload diseases, L1 remains one of the leading effective drugs for the treatment of such diseases. The reason for the effectiveness of this drug lies in several factors, including high affinity for iron ions, low redox activity of iron chelate complexes and also, high solubility and bioavailability [118,120,144,145,146].

Previous studies have shown that L1 has a stronger affinity (stability constant, log β), for Fe^3+^ (35.0), as well as to Cu^2+^ (19.6), than to other essential metals, such as Zn^2+^ (13.5). This illustrates the selectivity of L1 in relation to iron in comparison to the binding of other transition metal ions [105,118,146].

A major factor related to the toxicity of chelating drugs is the low redox activity of their metal complexes [3,147]. This property has been confirmed by both physicochemical studies in vitro, including the study of the electrode potential of chelate complexes of L1 using in electron paramagnetic resonance (EPR), nuclear magnetic resonance (NMR) and chemically induced dynamic nuclear polarization (CIDNP) and also the effect of its complexes with iron and copper ions in dark and photoinduced reactions, as well as in other in vivo studies [88,89,148,149,150,151]. In particular, it was shown that L1 inhibits the Fenton reaction with iron and copper ions as shown in Equations (1)–(8), reducing the yield of hydroxyl radicals to negligible or almost zero levels [88,147,148]. In addition, NMR studies of the effect of L1 on the peroxidation of linoleic acid (LA) micelles with the participation of iron and copper ions were also carried out. The results have shown that L1 is able to slow down the LA peroxidation reaction by more than 1000 times, which indicates a high antioxidant activity as a chelator used to treat diseases associated with an excess or catalytic iron in the body [88,89,147]. Furthermore, L1 has also shown antioxidant activity in the photochemical decomposition of iron aqua complexes with the formation of hydroxyl radicals [148]. In the case of therapeutic use for the treatment of patients with β-thalassemia, L1 also showed itself as a drug that reduces oxidative stress and restores the functions of different organs and tissues [151].

One of the main reasons for the redox inactivity of chelate complexes L1 is the low level of the electrode potential (−0.62 V for Fe^3+^/Fe^2+^ L1_3_) [86,150]. An additional factor influencing the antioxidant activity of L1 is the complete binding of iron and copper. As a bidentate chelator, L1 forms complexes with Fe^3+^ and Cu^2+^ with stoichiometry of 3:1 and 2:1, respectively, under physiological conditions [118,146,152,153]. Therefore, it is difficult for various oxidizing ligands, such as H_2_O_2_, to integrate into the internal coordination sphere for further oxidation reactions with a complexed metal ion.

Many other properties and characteristics contribute to the high efficiency of L1 as a therapeutic agent for the treatment of different diseases. These include the neutral charge of L1 and its iron complex at physiological pH, high solubility in water, hydrophilicity (partition coefficient n-octanol/water 0.19), high bioavailability, rapid gastrointestinal absorption (0.7–32 min) and elimination time of chelate complexes with iron ions (47–134 min) [105,118,154,155].

The influence of ascorbic acid on the redox activity of L1 at different concentrations was also investigated by EPR, NMR and UV-visible spectroscopy. It was found that, when the concentration of L1: Fe^3+^ is less than 2:1, ascorbic acid exhibits pro-oxidant effects and increases the production of ^•^OH in the Fenton reaction Equations (1)–(8). However, at higher concentrations of L1, ascorbic acid exhibits antioxidant activity and decreases the level of ^•^OH production. This phenomenon is explained by the incorporation of AscH_2_ into the inner sphere of the chelate complex [L1_3_Fe^3+^], replacing one of the L1 molecules, and forming a mixed complex [AscL1_2_Fe^3+^] with even lower redox activity than [L1_3_Fe^3+^] [149].

Despite that L1 has a high antioxidant activity and can be used to treat iron and copper overload diseases, such as thalassemia, various forms of siderosis, Friedeich’s ataxia, diabetes, Alzheimer’s disease, etc. [143,145,154,156,157,158,159,160], the chelator can also exhibit pro-oxidant activity under certain conditions. The mechanism proposed for the pro-oxidant activity of L1 involves a reaction between the chelator and the Fe^2+^ ion present mainly in iron-sulfur clusters [81]. In this context the complexed iron ion transfers its electron to the chelator, which in turn transfers an electron to oxygen with the formation of O_2_^•−^ and starts a cascade of redox reactions oxidizing various biomolecules, including aconitase-an iron-containing enzyme [161]. A confirmation mechanism of the pro-oxidant activity of L1 has also been suggested in a similar study [162]. It appears that the chelate complexes of L1 with Fe^2+^ are considered unstable and decompose to form Fe^3+^, which in turn is chelated by L1, giving inactive redox complexes [163]. It is noteworthy that no oxidation products are observed in this reaction, which means that L1 is not consumed [163]. The pro-oxidant activity of L1 within the mitochondria and its effect on mitochondrial metabolism has been proposed as the mechanism against cancer cells and an additional pathway promoting L1 for the treatment of cancer [160,161,162].

Despite the effectiveness of L1, and the preparation/screening of about 100 other KHP, work is in progress to synthesize more new chelators of the KHP group [118,120,122]. In this context and as originally proposed back in 2003, one of the main areas in developing chelating drugs is that of the treatment of neurodegenerative diseases such as Alzheimer’s disease, Parkinson’s disease and Friedreich’s ataxia [143]. Alzheimer’s disease is one of the most common neurodegenerative diseases leading to memory impairment and irreversible dementia. The lack of effective treatment methods and the decrease in human life expectancy make this disease a global public health problem [164]. The pathogenesis of Alzheimer’s disease is not fully understood, but there are several factors that play an important role in the pathophysiology of this disease, including the presence of extracellular deposits of amyloid plaques (Aβ), the accumulation of transition metal ions such as Fe^3+^ and Cu^2+^ and the oxidative stress caused by them, with uncontrolled generation of ROS and the dysfunction of several important neurotransmitters, in particular acetylcholine (ACh) depletion [165,166]. Thus, there are many targets and approaches for the development of new drugs for the treatment of Alzheimer’s disease.

There are a fairly large number of single-target investigational new drugs (IND), which have been proposed and tested for Alzheimer’s disease, but none has been found or shown to combat the causes and symptoms of the disease at the same time. Within this context, INDs are being synthesized that theoretically can act simultaneously on several targets in Alzheimer’s disease. Several structures of multifunctional compounds have been synthesized on the basis of L1, which, in addition to chelating and antioxidant activities, are also capable of affecting other targets of Alzheimer’s disease, such as inhibition of amyloid plaque deposits (anti-Aβ), inhibition of AChE and an increase in key neurotransmitters due to antagonistic activity histamine H3 receptor, as presented in Table 2:

All of the above chelators have antioxidant properties due to the presence of the L1-like 3-hydroxypyrid-one metal binding site in the structure of the molecule [115,142,172,173]. However, the greatest antioxidant activity is observed in the group of bi-tert-butyl-hydroxytoluene-containing chelators, which are antioxidant scavengers [115,139].

## 6. Anthraquinone Chelators

**Figure 5:**
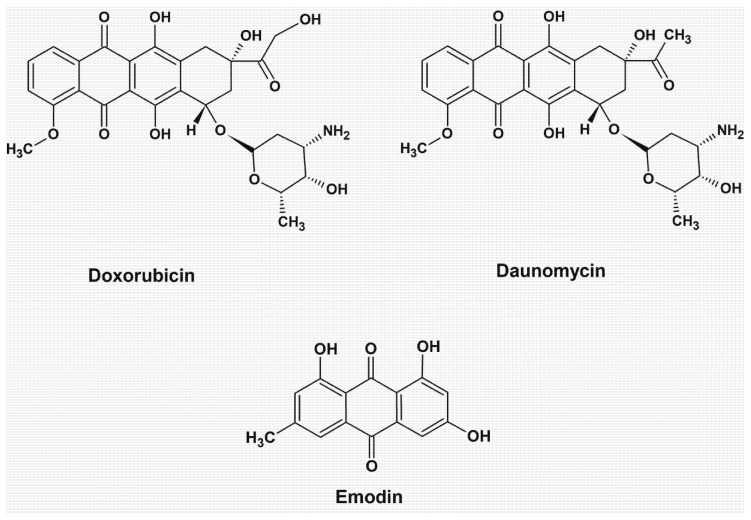
Chemical structures of the quinone-chelators doxorubicin (adriamicin, Dox), daunomycin (daunorubicin) and emodin.The metal binding sites of anthraquinones involves an oxygen molecule (=O) of one of the rings and an hydroxyl (−OH) group from an adjacent ring.

Anthraquinones, namely quinones of the anthracycline group, are mostly synthetic compounds, some of which belong to quinone-chelators. A feature of some of these compounds is their high antitumor activity. Some quinone-containing drugs/anthracycline antibiotics including doxorubicin (Dox), daunomycin and emodin (Figure 5) have become integral components of therapy regimens for various types of tumors, such as leukemia, lymphoma and breast cancer. The main cytotoxic effect of anthraquinones is to disrupt DNA synthesis, including inhibition of topoisomerase II. In addition, anthraquinones undergo cyclic reduction/oxidation reactions, accompanied by the release of ROS in biological electron transfer systems. However, the action of free radicals is also considered responsible for the cardiotoxic effect inherent in this class of compounds [135].

There are various reports on the role of chelation by quinones and other chelating agents in the generation of free radicals and in particular the generation of hydroxyl radicals, as well as their effect on lipid peroxidation [174,175,176,177,178,179]. Some of these data, which are mostly obtained from in vitro studies, describe the mechanisms of the effect of chelators in model reactions in solutions, including the effect of chelation on lipid peroxidation in liposomes and micelles [176,177,179]. While a significant part of the work in this area is devoted to the inhibition of lipid oxidation using various chelators [175,176,177], the major feature of quinone chelators is the enhancement of the generation of free radicals in reactions with metal ions [178,180].

Quinone chelators in the absence of metal ions are themselves capable of generating free radicals and affecting the rate of lipid oxidation [176,181]. Although the main mentioned mechanism and mode of action in anticancer therapy related to quinone chelators is DNA binding, the possibility of the existence of additional factors associated with the effect of quinone chelators on the oxidation of unsaturated lipids on the cell membrane has also been suggested [179].

Redox reactions with the participation of anthracycline quinone chelators are usually initiated by the reduction of a quinone molecule with the formation of a semiquinone radical. In this context, the cytochrome P450 enzyme system and the mitochondrial electron transfer chain are among the main sources for the one-electron reduction of quinone. The resulting semiquinone radical can then donate one electron to molecular oxygen, reducing it to the superoxide anion radical, and the semiquinone radical is oxidized back to the original quinone molecule. Thus, this process forms a redox cycle leading to the formation of superoxide anion radical and secondary reactive oxygen species (hydrogen peroxide, hydroxyl radical and peroxynitrite) [182].

Superoxide radical and hydrogen peroxide can generate highly reactive hydroxyl radicals in the Fenton reaction, which is catalyzed by transition metal ions [135]. Due to the large number of mitochondria in the myocardium, this mechanism is considered as the main cause of cardiotoxicity of anthracycline antibiotics. In particular, it was also found that there was an increase in the level of ROS after treatment with Dox both on cell lines and in animals. Biomarkers of oxidative stress are also elevated in human cancer patients treated by Dox. The most widely known mechanism of this observation is the ability of Dox to participate in redox reactions with the formation of ROS [137]. Some studies associate the cardiotoxicity of anthracyclines, and in particular Dox and daunomycin, with the ability to initiate the release of iron from the iron-storage protein ferritin [183]. It was argued that, in the presence of NADPH-cytochrome P-450 reductase, these drugs undergo a redox cycle with the formation of superoxide, which provides a slow release of iron from ferritin. In this case, the rapid release of iron from the ferritin is observed under anaerobic conditions, when there is no reaction of the semiquinone radical with oxygen. It was concluded that the semiquinone radicals of Dox and daunomycin are capable of directly releasing redox active iron that was previously deposited in ferritin [183].

However, there are studies indicating a different role for anthracyclines in iron metabolism. In particular, it was shown that Dox inhibits the release of iron from ferritin, which leads to accumulation and deposition of iron ions in this protein [183]. The authors attributed the difference of these conclusions from the results obtained on intact cell systems, while the work was carried out with the isolated protein ferritin [183]. It was suggested that the structure and functions of isolated protein may be different to the cellular protein in the cellular environment [184].

Another possible mechanism of anthracyclines cardiotoxicity is associated with their role in lipid peroxidation reactions. In particular, in vivo studies have shown that the yield of malondialdehyde, one of the markers of lipid peroxidation, in the cardiac tissues of experimental animals increases significantly in the presence of Dox [185]. It has also been shown that this quinone induces the formation of conjugated dienes as a result of lipid peroxidation in cardiomyocyte cells in vitro, which is accompanied by subsequent disruption of membrane integrity [186]. It appears that the release of iron from ferritin can lead to an increase in the fraction of free intracellular Fe^2+^, the subsequent formation of hydroxyl radicals, which can then initiate lipid peroxidation [183].

Quinones of the anthracycline group are widely used in medicine not only as highly effective cytotoxic agents in tumor chemotherapy, but also in photodynamic therapy. This approach makes it possible to locally activate the anticancer agent, increasing the specificity of the drug, and, thus, reducing the overall toxic side effects. Photodynamic therapy is a form of cancer treatment that is highly selective for tumor cells. The essence of this therapy is the formation of singlet oxygen and free radicals in the reaction of a photosensitizer with light of a suitable wavelength [187,188]. Singlet oxygen is a highly reactive molecule that oxidizes carbon-carbon bonds and can damage various components of the target cell (lipids, proteins, nucleic acids), which directly leads to the death of the tumor cell [188]. Photodynamic therapy of skin diseases is especially relevant due to the accessibility of tumor-affected areas to light [189]. However, it can also be used to treat other types of tumors by bringing thin light guides to the affected tissues [190]. Therefore, the search of effective agents is also quite relevant for photodynamic therapy.

Transition metal complexes that absorb light in the range suitable for photodynamic therapy (500–800 nm) have also been studied and shown to exhibit high cytotoxic activity against tumor cells of various histological origin [178,191,192,193]. Similarly, complexes of anthraquinones with transition metal ions demonstrated higher cytotoxicity under the influence of light (IC50~2–11 μM) in comparison to only anthraquinones, and also low cytotoxicity under dark conditions (IC50 > 50 μM) [193]. The enhancement of photo-cytotoxicity in the chelate complex with a copper ion appears from quantum-chemical calculations to occur due to the formation of a low-lying long-lived triplet state, which enhances the quinone’s ability to generate singlet oxygen by the energy transfer mechanism [193].

One of the first features of quinone chelate complexes is the appearance of a new light absorption band in the visible region (500–800 nm), which is more suitable for photodynamic therapy due to deeper penetration into living tissues [194]. In comparison, the light absorption region of the starting quinones lies in the UV range of 300–500 nm [195,196].

**Figure 6:**
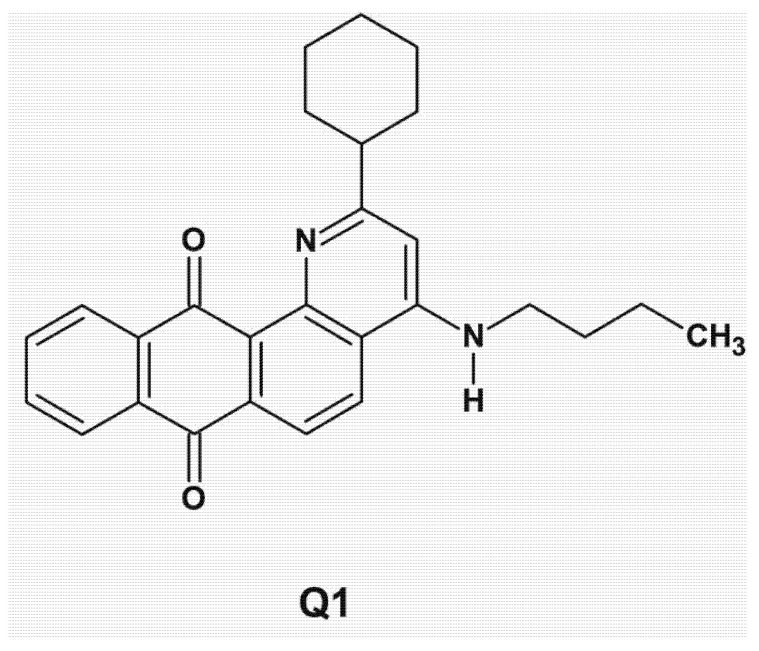
Chemical structure of the athraquinone-chelator 2-phenyl–4-(butylamino)naphtho[2,3-h] quinoline-7,12-dione (Q1).

Another feature of photoinduced processes involving chelate complexes of quinones is an increase in the yield of semiquinone radicals and ROS [178,180]. An increase in the yield of hydroxyl radicals in the presence of iron and zinc ions has been shown using EPR with spin traps in the presence of the quinone-chelators 2-phenyl–4-(butylamino)naphtho [2,3-h] quinoline-7,12-dione (Q1) and doxorubicin (Figure 5 and Figure 6) [178].

The formation of a semiquinone radical (Q^−•^) is a prerequisite for the formation of ROS upon reduction of quinones in the dark and upon excitation with visible light as shown in Equations (3), (12)–(16) [180]. The most likely biological electron donors for quinone molecules are glutathione (GSH) and the coenzyme NADH, which are present in cells at high concentrations [180,197,198].

(12) $$Q+e\to {\dot{Q}}^{-}$$

(13) $${\dot{Q}}^{-}+O_{2}↔Q+{\dot{O}}_{2}^{-}$$

(14) $${\dot{O}}_{2}^{-}+{\dot{O}}_{2}^{-}\overset{2H^{+}}{\to }H_{2}O_{2}+O_{2}$$

(15) $${\dot{O}}_{2}^{-}+H_{2}O_{2}\to O_{2}+{\mathrm{HO}}^{-}+H\dot{O}$$

(16) $${\dot{Q}}^{-}+{\mathrm{Fe}}^{3+}\to Q+{\mathrm{Fe}}^{2+}$$

In addition, ascorbic acid can also react with anthraquinones and their chelate complexes with Fe^2+^ ions as an electron donor as shown in Equations (17) and (18) [97,199].

(17) $$Q+{\mathrm{AscH}}^{-}\;\left({\mathrm{or}\;\mathrm{AscH}}_{2}\right)\to \dot{\mathrm{QH}}+{\dot{\mathrm{Asc}}}^{-}\left(\mathrm{or}\;{\dot{\mathrm{AscH}}}^{-}\right)$$

(18) $${\mathrm{QFe}}^{2+}+{\mathrm{AscH}}^{-}\;\left({\mathrm{or}\;\mathrm{AscH}}_{2}\right)\to {\mathrm{Fe}}^{2+}{\dot{Q}}^{-}+H^{+}+{\dot{\mathrm{Asc}}}^{-}\left(\mathrm{or}\;{\dot{\mathrm{AscH}}}^{-}\right)$$

Thus, ascorbic acid generates an additional channel for the reaction leading to ROS formation in the presence of anthraquinones [97,98,199]. This suggests the possible use of a combination of anticancer therapy with the participation of anthraquinones and ascorbic acid [97,98,200,201].

An increase of the yield of semiquinone radicals, as well as the NADH radical cation, was demonstrated in a model photoreaction in solution by the EPR and CIDNP methods in the presence of metal ions [178,180].

The principal property of the proposed mechanism in the reaction Equations (3), (12)–(16) is redox cycling (as a result of cyclic reduction of quinone), followed by the next oxidation of semiquinone with molecular oxygen. The Fenton reaction with the generation of a hydroxyl radical can take place not only in the presence of iron ions Equations (3), (12)–(16), but also in the presence of copper ions [88]. Due to their high reactivity and short lifetime, hydroxyl radicals must be formed in the vicinity of potential targets, thus causing local damage. The cyclic nature of the process should permit the inactivation of multiple copies of the target, thus allowing the use of low doses of reagent, with a concomitant reduction in toxic side effects. It should be noted that the detected increase in the yield of semiquinone radicals and ROS in the presence of metal ions was observed only for quinones-chelators but was absent for quinones that do not contain chelating groups [178,180].

An additional factor of the influence on the efficiency of electron transfer processes with the participation of quinones-chelators is the differential effect of metal ions such as Zn^2+^, Ca^2+^, Cu^2+^ and Fe^3+^ on the reduction potentials of quinones, for example Q1 and 1-amino-4-hydroxy-9,10-anthraquinone [178,198]. The increase of the electrode potential of quinone Q1 was about 0.5 V for the Zn^2+^, Ca^2+^, and Fe^3+^ complexes. Since non-transition metal ions do not participate in the generation of ROS by the Fenton reaction, it can be assumed that the increase of the quinone reduction potential effects the increase of the yield of semiquinone radicals in the presence of zinc ions as recorded by the EPR method [178] (see Scheme 1). A significant effect of complexation with the Cu^2+^ ion on the electrochemical behavior of 1-amino-4-hydroxy-9,10-anthraquinone, and in particular the stabilization of semiquinone, as a result of the redistribution of the electron density under the influence of the positive charge of the metal ion has also been noted [198]. It was suggested that this effect could reduce the ability of the semiquinone in complex with the copper ion to interact with molecular oxygen. Thus, in the case of the copper complex, there is a general decrease in the formation of O2^•−^ compared to free 1-amino-4-hydroxy-9,10-anthraquinone. It can be suggested from this study that the copper chelate complex will be less cardiotoxic than the parent quinone and can be considered for possible use in the treatment of cancer.

The effect of enhancing the activity of Dox in the presence of metal ions (Mg, Mn, Co, Ni, Fe, Cu, Zn) was also studied in vitro [195]. It was shown that Dox forms complexes with quinone-metal stoichiometry of 2:1 with bivalent metals, and 3:1 with Fe^3+^. A high antitumor activity of chelate complexes has been shown in the MCF-7 breast cancer cell model. Overall, it was concluded that selective quinone-metal complexes can be considered as new potential anticancer agents with higher efficiency than free quinones. However, further toxicological studies are needed, including studies of the molecular mechanisms of action of such complexes and possible changes in the signaling pathways of oxidative stress under the influence of the test compound.

## 7. Thiosemicarbazone Chelators

**Figure 7:**
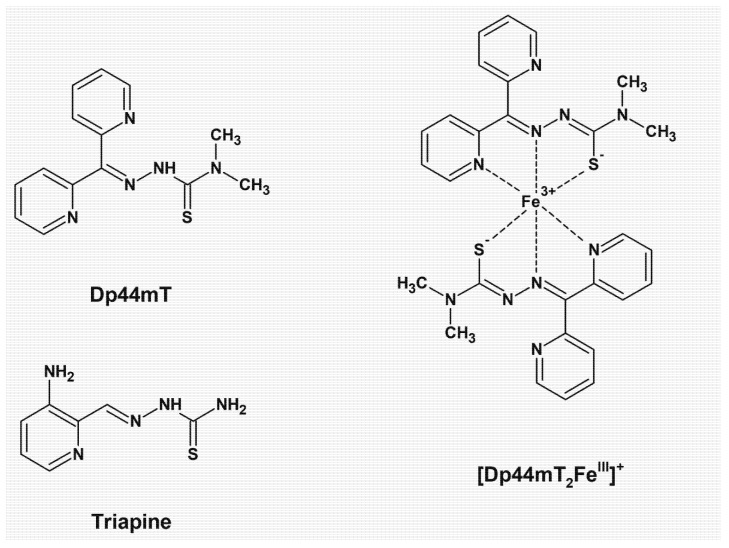
Chemical structures of 3-aminopyridine-2-carboxaldehyde thiosemicarbazone (triapine), di-2-pyridylketone-4,4,-dimethyl-3-thiosemicarbazone (Dp44mT) and its chelate complex with iron ion.

Thiosemicarbazones is another class of redox-active chelators which are of a great interest for scientists from various fields of science. In the PubMed database, a sharp surge in the number of publications devoted to these compounds is observed since the mid-2000s. The high interest stems from the wide range of their chemical and biological activity including their antimicrobial, antiviral and antitumor effects [202,203,204,205]. Some of the TSCs, such as triapine, have reached the stage of clinical trials in different categories of cancer patients, with encouraging anticancer effects [28,206,207,208,209,210] (Figure 7). The anticancer activity of TSCs was initially associated with the ability to inhibit ribonucleotide reductase [28,206,207,208,209,210,211]. Ribonucleotide reductase is an iron containing enzyme that converts all four ribonucleotides into their deoxyribonucleotide analogs, which plays an important role in DNA synthesis. Iron is of great importance for the catalytic activity of ribonucleotide reductase and stabilizes the tyrosyl radical located in one of the enzyme subunits. This makes iron a possible target for chemotherapy drugs [212].

The ability of TSCs to chelate metal ions is currently recognized as one of the main factors in their antiproliferative action at least by some investigators [213]. The redox activity of the complexes of TSCs with iron is of decisive importance in their anticancer activity, which is related to oxidative damage and inhibition of ribonucleotide reductase. Furthermore, the chelation of transition metals and in particular intracellular iron, can lead to inhibition of several other iron-containing proteins including ribonucleotide reductase [214]. Overall it appears that tumor cells have a higher level of ribonucleotide reductase than normal cells, and therefore are more sensitive to iron chelation [215]. In vivo studies and clinical trials have suggested that some TSCs can be developed and used as chemotherapeutic agents [28,206,207,208,209,210,215,216].

Recent studies have indicated a major role of redox activity and oxidative stress increase in the anticancer activity of TSCs [217]. A distinctive feature of the complexes of TSCs with iron is a rather high formal electrode potential of the Fe^3+/^Fe^2+^ pair, from +100 to +250 mV [213,217,218]. This leads to a higher stability of complexes with Fe^2+^ compared to complexes with Fe^3+^ [219]. In contrast, chelators used to treat diseases associated with excess iron, such as L1 and deferasirox, have a low redox potential of the Fe^3+/^Fe^2+^ pair, from −400 to −600 mV, which leads to a significantly higher stability of the complexes. with Fe^3+^ [219].

In general, Fe^2+^ in a complex with TSCs retains its redox catalytic activity. For example in the case the TSC chelator di-2-pyridylketone-4,4,-dimethyl-3-thiosemicarbazone (Dp44mT), Fe^2+^ is converted to Fe^3+^ in Dp44mT complexes via the reaction with molecular oxygen [220,221]. The proposed mechanism of the reactions proceeding in the buffer solution at pH 7.1 is shown in Equations (19)–(26) [221].

(19) $$[{\mathrm{Fe}}^{2+}{\left(\mathrm{TSC}\right)}_{2}]+H^{+}\overset{K_{H}}{↔}{\left[{\mathrm{Fe}}^{2+}{\left(\mathrm{TSC}\right)}_{2}H\right]}^{+}$$

(20) $$[{\mathrm{Fe}}^{2+}{\left(\mathrm{TSC}\right)}_{2}]+O_{2}\overset{k_{\mathrm{ox}}}{\to }{\left[{\mathrm{Fe}}^{2+}{\left(\mathrm{TSC}\right)}_{2}\right]}^{+}+{\dot{O_{2}}}^{-}$$

(21) $${\left[{\mathrm{Fe}}^{2+}{\left(\mathrm{TSC}\right)}_{2}H\right]}^{+}+O_{2}\overset{k_{\mathrm{ox}}}{\to }{\left[{\mathrm{Fe}}^{3+}{\left(\mathrm{TSC}\right)}_{2}\right]}^{+}+{H\dot{O}}_{2}$$

(22) $$[{\mathrm{Fe}}^{2+}]+{\dot{O_{2}}}^{-}+2H^{+}\to [{\mathrm{Fe}}^{3+}]+H_{2}O_{2}$$

(23) $$[{\mathrm{Fe}}^{2+}]+{H\dot{O}}_{2}+H^{+}\to [{\mathrm{Fe}}^{3+}]+H_{2}O_{2}$$

(24) $$[{\mathrm{Fe}}^{2+}]+H_{2}O_{2}+H^{+}\to [{\mathrm{Fe}}^{3+}]+\dot{O}H+H_{2}O$$

(25) $$2{\dot{O_{2}}}^{-}+2H^{+}\to O_{2}+H_{2}O_{2}$$

(26) $$2H_{2}O_{2}\to O_{2}+2H_{2}O$$

In the above equations, TSC is thiosemicarbazone and square brackets indicate chelate complex with various ligands in solution such as anions or water. Furthermore, since the complex of iron ion with TSCs is redox active, ascorbic acid is able to interact with it Equation (27) with the further formation of ROS Equations (20)–(26):

(27) $${\left[{\mathrm{Fe}}^{3+}{\left(\mathrm{TSC}\right)}_{2}\right]}^{+}+{\mathrm{AscH}}_{2}\left({\mathrm{or}\;\mathrm{AscH}}^{-}\right)\to \left[{\mathrm{Fe}}^{2+}{\left(\mathrm{TSC}\right)}_{2}\right]+\dot{\mathrm{AscH}}\left({\dot{\mathrm{Asc}}}^{-}\right)+H^{+}$$

This effect was demonstrated in studies using the EPR method with spin traps [222].

In the case of anthracycline antibiotics, there is an increase in the yield of the hydroxyl radicals in the Fenton reaction in the presence of TSCs, in particular the Dp44mT (Figure 7) [222]. A number of TSCs also demonstrate a significant increase in the yield of ROS in experiments in cell cultures of HCT116 (colon cancer), accompanied by cell death [216].

An important factor affecting the activity of TSCs is their hydrophobicity or lipophilicity. The octanol/water partition coefficient (logP) can be a measure of this factor. It has been shown that the most hydrophobic TSCs (logP > 2) and the most hydrophilic (logP < 1.5) demonstrate a worse antiproliferative effect than TSCs with logP 1.5–2.0 [213,217]. The probable reason is that the most hydrophilic compounds are difficult to pass through the cell membrane, while highly hydrophobic molecules will accumulate in the membranes due to their high affinity for the lipid environment.

**Figure 8:**
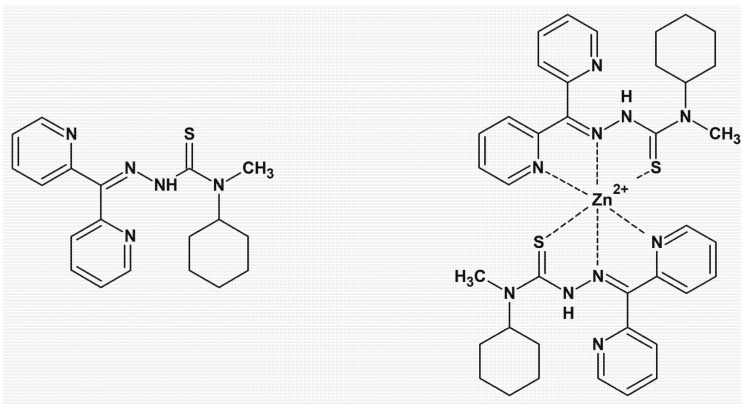
Chemical structures of di–2–pyridylketone–4–cyclohexyl–4–methyl–3-thiosemicarbazone (DpC) and its chelate complex with zinc ion.

Complexes of TSCs with copper and zinc also have an antiproliferative effect. Thus, the complexes of Dp44mT and di–2–pyridylketone–4–cyclohexyl–4–methyl–3-thiosemicarbazone (DpC) with zinc were found to exhibit higher cytotoxicity than free ligands (Figure 8) [223]. Microscopy with fluorescent labels showed that TSCs penetrate into lysosomes with the formation of copper chelate complexes, promoting the formation of ROS, membrane oxidation and cell death. It has been proposed that this mechanism of action is also related to the anticancer activity of TSCs [223,224].

**Figure 9:**
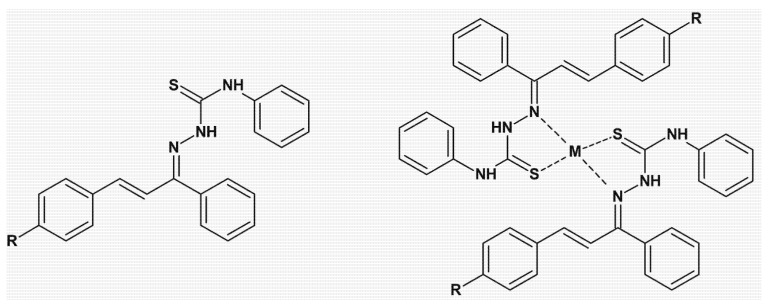
Chemical structures of 2-((E)–3-(4-chlorophenyl)–1-phenylallylidene)–*N*–phenyl-hydrazinecarbothioamide (HL1, R = Cl) and 2-((E)–3-(4-cyanophenyl)–1-phenylallylidene)–*N*–phenyl-hydrazinecarbothioamide (HL4, R = CN) and their complexes with metal ions (M).

However, this mechanism, which includes lipid peroxidation in membranes, is not characteristic of all compounds of this class. Thus, studies have shown that the complexes of 2-((E)-3-(4-chlorophenyl)-1-phenylallylidene)-*N*-phenyl-hydrazinecarbothioamide (HL1) and 2-((E)-3-(4-cyanophenyl)-1-phenylallylidene)-*N*-phenyl-hydrazinecarbothioamide (HL4) (Figure 9) with copper have an antiproliferative effect in experiments on *S. cerevisiae* cells [225]. However, difference in the mechanism of action has been proposed where, in the case of HL4, the effects are on proliferation by preventing the formation of viable daughter cells, whereas, in the case of HL1, the effects are related to the induction of lipid peroxidation in the plasma membrane [225].

It has also been shown that complex formation with copper leads to an increase in the selectivity of the TSCs against melanoma cells. For example, complexes of triapine with copper exhibit greater cytotoxicity in relation to tumor cells and less in relation to normal cells than the free ligand [226,227,228,229,230,231]. Copper accumulates in tumor cells due to the selective permeability of cancer cell membranes. In general it was found that many complexes of TSCs with copper are active against malignant tumors both in in vitro and in vivo tests [97,226,227,228,229,230].

## 8. Conclusions

The redox properties of chelating drugs and their metal complexes, which are used to treat various diseases, are one of the central factors in their therapeutic activity and a major aspect of their toxicity. In the case of oxidative stress caused by the catalytic activity of iron and copper, some chelators are used to inhibit the redox activity of these metal ions. Conversely, in some forms of cancer treatment, metal complexes are required, which have redox activity and can enhance the redox properties of bounded metal ions. The spectrum of chelator mechanisms that affect the redox activity of complexed metals is quite diverse and includes the ability of the chelator to participate in redox reactions, a change in the electrode potential of the metal in the complex and the creation/preservation of the coordination center for metal binding in the complex. In this review, the diversity of molecular properties and redox mechanistic insights of chelating drugs and chelator metal complexes with a wide range of potential therapeutic uses has been shown with emphasis three major prototype groups, namely the alpha-ketohydroxypyridines, anthraquinones, and thiosemicarbazones. Different therapeutic possibilities can be envisaged in this series of chelators including the possibility of modifying the basic chemical structure and adding functional groups, which can potentially lead to multi-target therapy of various pathologies, for example, in Alzheimer’s disease. In addition, the physicochemical and chelating properties of ascorbic acid are described, as well as the possibility of its use together with other chelating drugs or agents in combination therapies for the treatment of various pathologies. It should be noted that despite the progress and development in this area, the physicochemical, biological and toxicological properties of new chelators have not yet been fully investigated; additional in vitro, in vivo, and clinical studies are needed for the development of related therapeutics in cancer, neurodegenerative and other diseases.

## Figures and Tables

**Table 1:** Rate constants of the reaction of free radicals with ascorbic acid at pH 7.4.

Radical	k (M^−1^s^−1^)
^•^OH (hydroxyl)	1.1 × 10^10^
RO^•^ (tert-butyl alkoxyl radical)	1.6 × 10^9^
ROO^•^ (alkyl peroxyl radical, e.g., CH_3_OO^•^)	1–2 × 10^6^
GS^•^ (glutathiol radical)	6 × 10^8^
TO^•^ (tocopheroxyl radical)	2 × 10^5^
Asc^•−^ (dismutation)	2 × 10^5^
O_2_^•−^/^•^OOH	1 × 10^5^

**Table 2:** Chemical structures of alpha-ketohydroxypyridine hybrids as potential multi-target anti Alzheimer disease drugs.

Chelator		Types of Anti-Alzheimer Activity	References *
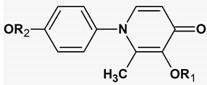	R_1_,R_2_ = H, 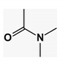	AoA AChE Abeta	[117]
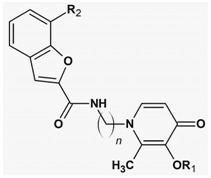	*n* = 2–4 R_1_ = H, Bn R_2_ = H, OCH_3_	AoA AChE Abeta	[167,168]
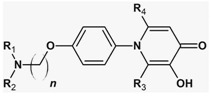	*n* = 3–5 NR_1_R_2_ = 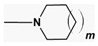 , m = 0,1 NR_1_R_2_ = 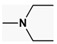	AoA Abeta H_3_Ra	[169,170]
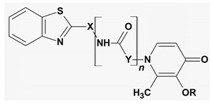	*n* = 1 X,Y = -CH_2_Ph, -Ph, -(CH_2_)_n_ *n* = 0 X = Ph R = H, Bn	AoA AChE Abeta	[117,171]
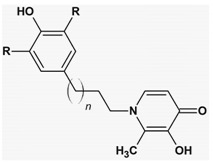	*n* = 1–3 R = *t*-Bu, OCH_3_	AoA	[139]

* Antioxidant activity (AoA), anti-acetylcholinesterase (AChE), anti-amyloid beta aggregation (Abeta) and H_3_ receptor antagonism (H_3_Ra).

## Data Availability

Data is contained within the article.

## Abbreviations

Abeta	anti-amyloid beta aggregation;
AChE	anti-acetylcholinesterase;
anti-Aβ	inhibition of amyloid plaque deposits;
AoA	antioxidant activity;
Asc^•−^	ascorbyl anion radical;
AscH_2_	ascorbic acid;
AscH^•^	ascorbyl radical;
AscH^−^	ascorbate anion;
CIDNP	chemically induced dynamic nuclear polarization;
Dox	Doxorubicin;
DpC	di-2-pyridylketone-4-cyclohexyl-4-methyl-3-thiosemicarbazone;
DHA	dehydroascorbate;
DHP	dihydropyridine;
DNA	deoxyribonucleic acid;
Dp44mT	di-2-pyridylketone-4,4,-dimethyl-3-thiosemicarbazone;
EPR	electron paramagnetic resonance;
GSH	glutathione;
HL1	2-((E)-3-(4-chlorophenyl)-1-phenylallylidene)-*N*-phenyl-hydrazinecarbothioamide;
HL4	2-((E)-3-(4-cyanophenyl)-1-phenylallylidene)-*N*-phenyl-hydrazinecarbothioamide;
H_2_O_2_	hydrogen peroxide;
H_3_Ra	H3 receptor antagonism;
IND	investigational new drugs;
KHP	alpha-ketohydroxypyridines;
LA	linoleic acid;
logP	octanol/water partition coefficient;
L1	deferiprone;
NADH	nicotinamide adenine dinucleotide;
NMR	nuclear magnetic resonance;
^1^O_2_	singlet oxygen;
OH	hydroxyl radical;
OOH	peroxyl radical;
Q1	2-phenyl-4-(butylamino)naphtho[2,3-h] quinoline-7,12-dione;
Q^−•^	semiquinone radical;
RNA	ribonucleic acid;
ROS	reactive oxygen species;
TSC	thiosemicarbazone;
UV-Vis	ultraviolet-visible.
